# RNA Interference of *Trypanosoma brucei* Cathepsin B and L Affects Disease Progression in a Mouse Model

**DOI:** 10.1371/journal.pntd.0000298

**Published:** 2008-09-24

**Authors:** Maha-Hamadien Abdulla, Theresa O'Brien, Zachary B. Mackey, Mohamed Sajid, Dennis J. Grab, James H. McKerrow

**Affiliations:** 1 Sandler Center for Basic Research in Parasitic Diseases, California Institute for Quantitative Biomedical Research, University of California San Francisco, San Francisco, California, United States of America; 2 Department of Pediatrics, Division of Infectious Diseases, The Johns Hopkins University School of Medicine, Baltimore, Maryland, United States of America; Yale University School of Medicine, United States of America

## Abstract

We investigated the roles played by the cysteine proteases cathepsin B and cathepsin L (brucipain) in the pathogenesis of *Trypansoma brucei brucei* in both an in vivo mouse model and an in vitro model of the blood–brain barrier. Doxycycline induction of RNAi targeting cathepsin B led to parasite clearance from the bloodstream and prevent a lethal infection in the mice. In contrast, all mice infected with *T. brucei* containing the uninduced *Trypanosoma brucei* cathepsin B (TbCatB) RNA construct died by day 13. Induction of RNAi against brucipain did not cure mice from infection; however, 50% of these mice survived 60 days longer than uninduced controls. The ability of *T. b. brucei* to cross an in vitro model of the human blood–brain barrier was also reduced by brucipain RNAi induction. Taken together, the data suggest that while TbCatB is the more likely target for the development of new chemotherapy, a possible role for brucipain is in facilitating parasite entry into the brain.

## Author Summary

African trypanosomiasis, or sleeping sickness, is caused by the single-cell parasite *Trypanosoma brucei* (*T. brucei*). Two parasite-derived enzyme proteins have been hypothesized to play an important role in the viability of the parasite or its ability to produce disease in the human host. Utilizing RNA interference that blocks the production of these proteins in the parasite, we show that elimination of parasite cathepsin B cures infection in mice. RNAi of the second enzyme protein, brucipain, results in the prolongation of life of half the infected mice, but does not cure. Further experiments carried out in a culture system show that brucipain facilitates the migration of parasites across a model of the blood–brain barrier. This suggests that while brucipain is not necessary for the viability of the organisms, it may play a role in infection by allowing parasites to reach the central nervous system and produce the severe second stage of sleeping sickness.

## Introduction

Subspecies of *Trypanosoma brucei* are the causative agents of human African trypanosomiasis. In vitro studies utilizing both small molecule cysteine protease inhibitors and RNA interference (RNAi) have implicated the Clan CA (papain) family of cysteine proteases as critical to the successful lifecycle of *Trypanosoma brucei brucei* (*T. b. brucei*) [1],[2]. In vivo studies have demonstrated that cysteine protease inhibitors prolong the lives of mice infected with lethal inocula of trypanosomes [1],[3]. There are two distinct Clan CA cysteine proteases identified in the *T. brucei* genome. Brucipain (aka trypanopain-Tb, rhodesain) is a cathepsin L-like protease responsible for the bulk of protease activity in the organism [2]. *Trypanosoma brucei* cathepsin B (TbCatB) is a more recently characterized protease that is upregulated in the bloodstream stage of the parasite [2]. In *in vitro* studies, RNAi of TbCatB produced swelling of the endosome compartment analogous to that seen with class-specific cysteine protease inhibitors [1],[2] and led to arrest of trypanosome replication and death. In contrast, knockdown of brucipain by RNAi in vitro produced no detectable phenotypic changes. However, it was hypothesized that this enzyme might play a role in the degradation of mistargeted glycosylphosphatidylinisotol (GPI) anchored proteins, VSG turnover, disruption of the blood–brain barrier, or degradation of host immunoglobulin [4],[5] While RNAi with cultured parasites can provide important insights into the role of a specific gene product in parasite replication and viability, a role in pathogenesis, as proposed for brucipain, can only be validated in vivo. We show that introduction of RNAi from a tetracycline-inducible promoter can be achieved in vivo in a mouse model of *T. b. brucei* infection, and show that transcriptional silencing of either of these two proteases alters the course of *T. b. brucei* infection [6].

## Materials and Methods

### Bloodstream *T. brucei* strain 90-13

Bloodstream *T. brucei* strain 90-13 was electroporated with plasmids containing either brucipain (TbRho), TbCatB, or GFP transgenes [2]. The plasmid used, pZJM, allows transfected organisms to be induced to produce RNAi in the presence of tetracycline. The brucipain RNAi construct used for this study is one of three partial open reading frames (ORF) of brucipain used to down regulate its message *in vitro*. R1 encodes a cDNA that corresponding to the first 597 nucleotides of brucipain ORF. R2 encodes a cDNA encoding the middle 400 nucleotides of the brucipain ORF and R3 encodes a partial cDNA encoding the last 300 nucleotides of the brucipain ORF. Each of these constructs were capable of efficiently and specifically silencing the mRNA of brucipain *in vitro.* The same *T. b. brucei* clones expressing the R1 construct used in a previous study [2]. The TbCatB transgene has been described in detail previously [2]. To generate the GFP transgene, the gene encoding GFP (714 nucleotides) was amplified from the pHD-HX-GFP vector [7]. Methods for electroporation and selection of stable transformants have been described [2].

### Trypanosome culture and infection of mice

Bloodstream form (BSF) 90-13 cells expressing T7 RNA polymerase and tetracycline repressor protein were maintained in HMI-9 medium [8]. Five BALB/c mice per group (6–8 weeks old) were infected by intraperitoneal injection with 600 parasites carrying pZJMTbRho, pZJMTbCatB, or pZJMGFP plasmids or with control 90-13 parasites. To rule out any direct effects of doxycycline on the course of trypanosome infection, two additional groups of mice were infected with the parental *T. b. brucei* strain 90-13. One group was given doxycycline-containing food (200-mg/Kg, Bioserv Corporation, San Diego, CA) and water containing 1 mg/ml doxycycline hyclate (Sigma-Aldrich), the second group was given standard food and water.

Six other groups of mice were infected with *T. b. brucei* containing an RNAi-producing plasmid for brucipain (pZJMTbRho), cathepsin B (pZJMTbCatB), or GFP (pZJMGFP). Three control (uninduced) groups were given standard food and water, and another three groups were given doxycycline containing food and water. The two groups infected with pZJMGFP served as a control for a gene that is not found in the trypanosome. Mice were monitored every other day for weight loss, general appearance, and behavior. Experiments were carried out in accordance with protocols approved by the Institutional Animal Care and Use Committee (IACUC) at UCSF.

### The *in vitro* model of the human blood–brain barrier (BBB)

We used a human brain microvascular endothelial cell (BMEC) line whose phenotypic expression was stabilized by immortalization with pSVT, a pBR322-based plasmid containing the DNA sequence encoding the simian virus 40 large-T antigen [9]. Similar to the primary human BMEC cell line (XIII) from which they were derived, the transfected human BMECs are positive for FVIII-Rag, carbonic anhydrase IV, and Ulex europeus agglutinin I; take up acetylated low-density lipoprotein; and express gamma glutamyl transpeptidase [9],[10]. Human BMECs were cultured at 37°C in medium 199 (GIBCO) supplemented with 20% heat-inactivated fetal bovine serum and 1× Glutamax (GIBCO) in a humidified environment of 95% air, 5% CO_2_. The cells were grown to confluence on 6.5-mm-diameter collagen-coated Costar Transwell inserts with a pore size of 3.0 m until transendothelial electrical resistance (TEER) measurements exceeded 25 cm^2^ [11]. For the transmigration study, the parasites were added to the top of the human BMEC-containing inserts. The cultures were incubated with and without tetracycline (100 ng/ml) in triplicate at 37°C, and the number of parasites present at the bottom chamber were determined by counting aliquots in the Neubauer chamber.

### Real-time reverse transcription RT-PCR

Gene transcripts for brucipain were quantified in freshly isolated *T. b. brucei* from mice infected with pZJMTbRho at five days post infection. Blood was separated in a DEAE-sepharose column as previously described [12]. Total RNA extraction from *T. b. brucei* was performed using the TRIzol reagent (Invitrogen, Carlsbad, CA). RT-PCR, the one-step RT-PCR kit (Invitrogen, Carlsbad, CA), and gene-specific primers forward 5′-ATACGCAACGTTTGGTGTGA-3′ and reverse 5′CCTTCGATGTTGCCGATAGT -3′ were used to amplify brucipain. The relative amount of gene transcripts was calculated using methods previously described [13].

### Preparation of trypanosome lysates

Parasites were purified from mice infected with parental 90-13 or pZJMTbRho. As reported previously [12], T. b. brucei from infected mice were harvested by centrifugation, washed once in PBS-containing 1% glucose, and resuspended in lysis buffer (1.0% Triton X-100, 10 mM Tris pH 7.5, 25 mM KCl, 150 mM NaCl, 1 mM MgCl2, 0.2 mM EDTA, 1 mM dithiothreitol, 20% glycerol). The lysates were incubated on ice for 20 min and cleared by centrifugation at 16,000 g for 15 min at +4°C. Protein concentration of was determined by the Bradford assay (Bio-Rad).

### Western blots of trypanosome lysates following RNAi induction

Ten µg of trypanosome lysate was resolved by 15% SDS-PAGE and transferred a to polyvinylidene difluoride (PVDF) membrane. After transferring and blocking, the PVDF membranes were incubated with rabbit anti-brucipain antiserum (1∶2500 dilution) or anti-TbcatB 1∶2000 [14] for 1 h and washed three times for five min with TBST (10 mM Tris, pH 7.4, 150 mM NaCl, 0.4% Tween 20). After the third wash, horseradish peroxidase-conjugated donkey anti-rabbit IgG (1∶1,000 dilution) was added to the blots for 1 h. The blots were washed again in the same buffer three times for five min and examined by ECL (Amersham Biosciences).

### Radiolabeling of cysteine protease active sites with ^125^I-labeled inhibitors

Equal amounts of trypanosome lysate (10 µg) were labeled with ^125^I-DCG-04 [15] in the presence of 2 mM dithiothreitol for 45 min at room temperature and subjected to SDS-PAGE. Quantification of labeled enzymes was determined by Phosphoimager analysis (Molecular Dynamics).

### Statistical analysis

Data were analyzed using the Mann-Whitney nonparametric test to determine the statistical difference in spleen weight in induced versus un-induced infected mice. Chi-square analysis was performed to determine the significant difference in survival.

## Results/Discussion

**Figure 1:**
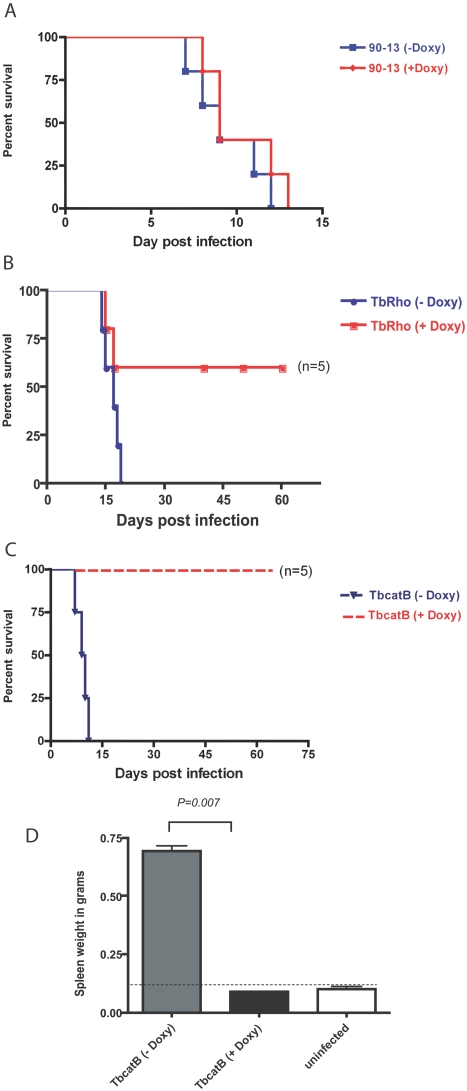
Survival analysis for mice infected with *T. b. brucei*. (A) Kaplan-Meier survival analysis for mice infected with *T. b. brucei* not transfected with the RNAi construct but given an inducing dose of doxycycline (red) versus standard food and water (blue) (n = 5 in each group). (B) Infection with trypanosomes containing the RNAi plasmid for brucipain plus or minus induction by doxycycline. Brucipain RNAi resulted in prolonged survival of three out of the five mice (p = 0.004) (experiment was conducted twice with the same result). (C) Infection with parasite containing RNAi plasmid for TbCatB plus or minus induction by doxycycline. Note that all five mice infected with parasites in which cathepsin B RNAi was induced survived until the experiment was terminated. (D) Spleen weights in mice infected with cathepsin B RNAi parasites and induced with doxycyline were within normal range compared to uninfected controls.

**Figure 2:**
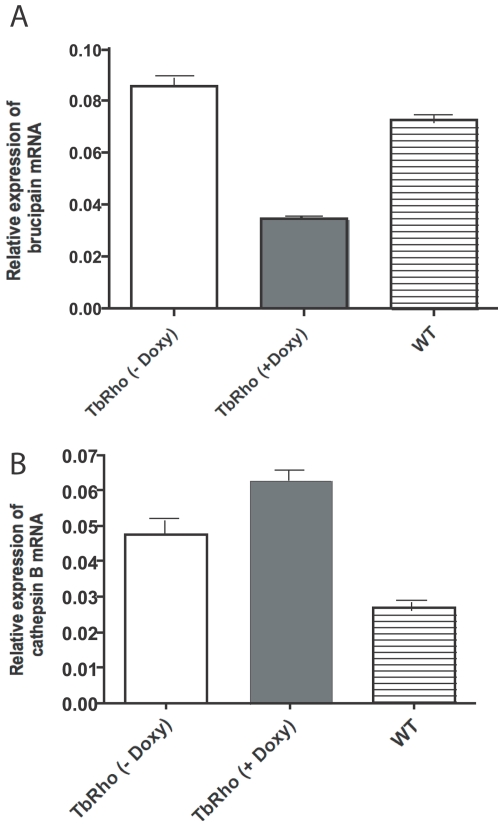
RNAi reduces expression of brucipain mRNA in parasites isolated from mice. (A) Evidence that RNAi reduces expression of brucipain mRNA in parasites isolated from mice. Mice infected with the brucipain RNAi-containing plasmids were either induced with doxycycline or left un-induced. (B) RNAi induced against brucipain did not decrease mRNA level for cathepsin B.

**Figure 3:**
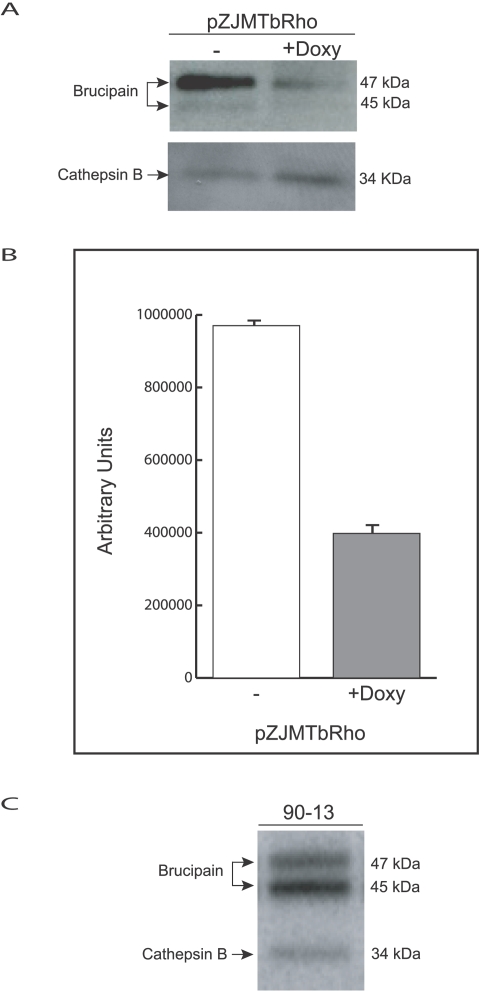
RNAi reduces brucipain protein and protease activity. (A) Equal amounts of protein were resolved by 15% SDS-PAGE, stained with anti-rhodesain antibody, and visualized by Western blot. Brucipain protein level is decreased after RNAi induction in pZJMTbRho parasites recovered from infected mice but the level of cathepsin B is not decreased after brucipain RNAi induction in pZJMTbRho parasites. (B) Brucipain activity is also decreased by 60% with brucipain RNAi induction. The level of brucipain activity in pZJMRho transfected parasites purified from mice was determined with the active site tag ^125^I-DCG-04, visualized by autoradiography, and quantified by PhosphorImager analysis. (C) In the absence of RNAi bands of brucipain and TbCatB activity can be identified in purified parasites from mice infected with 90-13 labeled with the active site tag ^125^I-DCG-04 and visualized by autoradiography.

The goal of these experiments was to validate the in vitro effects of RNAi on TbcatB in an in vivo disease model of African trypanosomiasis, and to explore a potential role of brucipain as a virulence factor. For safety reasons we conducted the knockdown experiment in the human non-infective strain T. b. brucei which has been traditionally grown and studied in mice. Doxycycline by itself produced no significant alteration (+/−1 day) in the course of *T. b. brucei* 90-13 infections (Fig. 1A). Equivalent levels of parasitemia and splenomegaly were observed in mice whether or not they were maintained on a doxycycline-containing diet (not shown). The in vivo induction of RNAi against brucipain in *T. b. brucei* did not cure infection, but extended the survival of three out of five mice beyond 60 days (Fig. 1B) the experiment was repeated twice with the same result. All mice infected with trypanosomes having the brucipain transcript knockdown had parasitemia and splenomegaly equivalent to that seen in control mice at the time of their sacrifice (not shown). Splenomegaly (quantified by spleen weight) is a convenient gross pathological marker of disease burden [16]. Analysis of mRNA levels in trypanosomes isolated from infected mice confirmed 60% reduction in the level of brucipain mRNA (Fig. 2A). The level of cathepsin B mRNA was not affected by RNAi induction against brucipain in pZJMTbRho induced parasites (Fig. 2B). Active site labeling of brucipain in trypanosomes purified from mouse blood confirmed 60% reduction in brucipain protease activity (Fig. 3C). Endogenous activity levels of brucipain and cathepsin B, quantified by DCG-04 labeling of purified parasites from mice infected with 90-13 strain, confirmed that brucipain was more abundant than cathepsin B (Fig. 3D), consistent with previously published data [2],[14]. A control cell line with an insert of GFP was generated to investigate the role of RNAi plasmid construct itself on the parasites in vivo. No difference was seen in mouse pathology or in brucipain or cathepsin B levels with GFP-induced parasites (data not shown).

In vivo induction of TbCatB RNAi resulted in survival of all five mice for up to two months post infection, after which time the experiment was terminated (Fig. 1C). Un-induced mice began to die 13 days after infection. No trypanosomes were detected in the blood of mice infected with pZJMTbCatB trypanosomes after induction of RNAi with doxycycline. These mice also had normal spleen weights compared to un-induced controls (Fig. 1D). Control mice with no doxycycline died between day 11 and 15 post infection. The last day on which untreated mice died from the trypanosome infection may vary depending on the exact parasite inoculum received and other host defense and host metabolic factors (Fig. 1A vs 1B).

The demonstration that doxycycline induction of RNAi can be achieved in parasites within an animal model of infection is an important technological advance that should encourage the use of this approach by other investigators. The failure of parasites to establish infection with TbCatB RNAi might have been predicted from *in vitro* assays. However demonstration in an *in vivo* model of infection is a significant and necessary validation of the key role of TbCatB in infection. The effect of reducing transcripts for the cathepsin L-like trypanosome protease (brucipain) on the progression of the infection was not predicted from *in vitro* assays. The effect of brucipain RNAi suggests that the cathepsin L protease might play a role as a virulence factor in *in vivo* infections, where host tissue tropism and the host immune response add new layers of complexity.

In conclusion, gene-specific RNAi can be induced in bloodstream parasites in an experimental model of trypanosome infection. Induction of RNAi targeting TbCatB transcripts in vivo correlates with the results observed in previous in vitro RNAi experiments [1],[2]. In the mouse model of infection, RNAi of TbCatB rescued mice from a lethal *T. b. brucei* infection, resulting in no splenomegaly and no detectable parasites in blood. While induction of RNAi against brucipain in two independent experiments did not cure mice of their infection, it did significantly prolong the survival of five out of ten mice. Since RNAi led to a 60% reduction of brucipain activity (Fig. 3C), it is still possible that a 100% knockdown might uncover a more direct role for brucipain in parasite viability; brucipain knockouts are being pursued as strategy to more clearly delineate the role of brucipain. However, even the modest RNAi knockdown achieved for TbCatB (quantified in [2]) had a profound negative effect on parasite viability both in vitro [5] and in vivo, suggesting that *T. brucei* cathepsin B is the more likely target for protease inhibitors as chemotherapy against human African trypanosomiasis [17].

**Figure 4:**
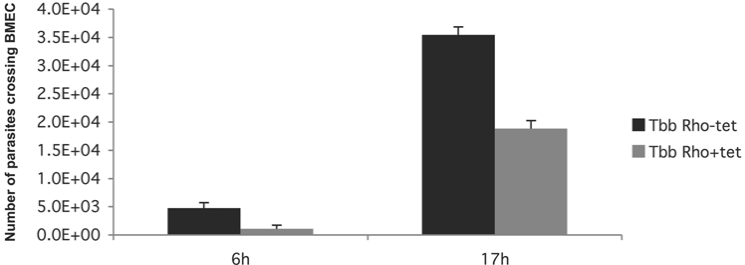
Parasite traversal across human BMEC. Transwell inserts containing human BMEC (initial TEER = 26.3 Ω) were incubated with 3×10^5^ pZJMTbRho RNAi trypanosomes (+/−tetracycline) and the number of parasites that crossed the BMEC monolayers into the bottom wells determined. All values represent the mean±SEM of triplicate determinations.

While the residual brucipain activity seen after RNAi induction might be responsible for disease progression in two of the mice shown in (Fig. 1B), an alternative conclusion is that brucipain plays a specific role in Trypanosoma pathogenesis in vivo, but not in parasite viability per se. Nikolskaia et al. [5] showed that a cysteine protease inhibitor, known to target brucipain, blocked the ability of African trypanosomes to cross a model of the blood–brain barrier (BBB) [5]. Using this in vitro model of the blood–brain barrier, we confirmed that brucipain is required for African trypanosomes to effectively cross the brain endothelial barriers. Without tetracycline 3.54E+04±1.41E+03 (mean±SEM) of the initial brucipain RNAi trypanosome (pZJMTbRho–tet) inoculum crossed the endothelial cell barrier (1–2%) (Fig. 4). This is comparable to those noted for *T. b. brucei* 427 and TREU 927 in previously published reports [5],[11]. However when brucpain RNAi was induced by tetracycline, the number of parasites migrating across the barrier was reduced by 50% (1.10E+03±6.35E+02), (*p* = 0.003). The human BMEC transendothelial electrical resistance (TEER) at the end of the experiment was 30.4±1.2 ohms (*p* = 0.00002), indicating that barrier integrity was maintained for all *T. b. brucei* treatment conditions. To rule out any effect of tetracycline on the in vitro BBB model other than induces RNAi, trypanosomes (pZJMTbRho) were pretreated with tetracycline, but the antibiotic was then removed and the parasites incubated with human BMEC overnight. The number of parasites crossing the BMEC was the same as control (with tetracycline), demonstrating that tetracycline has no effect on endothelial cells (data not shown). Experiments were repeated twice with the same result. In summary, the data show that knockdown of brucipain transcripts by RNAi led to reduced protease activity but no effect on parasitemia or splenomegaly. However the prolonged survival of some of the infected mice might be due to inability of the parasite to efficiently enter the CNS.
